# Surveillance of endemic coronaviruses during the COVID‐19 pandemic in Iran, 2021–2022

**DOI:** 10.1111/irv.13128

**Published:** 2023-03-24

**Authors:** Hassan Karami, Kaveh Sadeghi, Sevrin Zadheidar, Fatemeh Saadatmand, Negar Mirsalehi, Nima Hoveidi Ardestani, Shirin Kalantari, Mohammad Farahmand, Jila Yavarian, Talat Mokhtari‐Azad

**Affiliations:** ^1^ Department of Virology, School of Public Health Tehran University of Medical Sciences Tehran Iran; ^2^ Research Center for Antibiotic Stewardship & Antimicrobial Resistance Tehran University of Medical Sciences Tehran Iran

**Keywords:** COVID‐19, HCoV‐229E, HCoV‐HKU1, HCoV‐NL63, HCoVs‐OC43, Iran

## Abstract

**Background:**

Human coronaviruses (HCoVs) 229E, OC43, HKU1, and NL63 are common viruses that continuously circulate in the human population. Previous studies showed the circulation of HCoVs during the cold months in Iran. We studied the circulation of HCoVs during coronavirus disease 2019 (COVID‐19) pandemic to find the impact of pandemic on the circulation of these viruses.

**Methods:**

As a cross‐sectional survey conducted during 2021 to 2022, of all throat swabs sent to Iran National Influenza Center from patients with severe acute respiratory infection, 590 samples were selected to test for HCoVs using one‐step real‐time RT‐PCR.

**Results:**

Overall, 28 out of 590 (4.7%) tested samples were found to be positive for at least one HCoVs. HCoV‐OC43 was the most common (14/590 or 2.4%), followed by HCoV‐HKU1 (12/590 or 2%) and HCoV‐229E (4/590 or 0.6%), while HCoV‐NL63 was not detected. HCoVs were detected in patients of all ages and throughout the study period with peaks in the cold months of the year.

**Conclusions:**

Our multicenter survey provides insight into the low circulation of HCoVs during the COVID‐19 pandemic in Iran in 2021/2022. Hygiene habits and social distancing measures might have important role in decreasing of HCoVs transmission. We believe that surveillance studies are needed to track the pattern of HCoVs distributions and detect changes in the epidemiology of such viruses to set out strategies in order to timely control the future outbreaks of HCoVs throughout the nation.

## 1 INTRODUCTION

Human coronaviruses (HCoVs) are pathogenic viruses of humans with a high zoonotic potential.^1^ These viruses are believed to be originated and evolved in animals and known to be characterized by a crown‐like appearance and a large, nonsegmented, positive, single‐stranded ribonucleic acid.^2^ There are seven strains of HCoVs infectious to humans recognized as HCoV‐OC43, HCoV‐NL63, HCoV‐HKU1, HCoV‐229E, severe acute respiratory syndrome coronavirus (SARS‐CoV), Middle East respiratory syndrome coronavirus (MERS‐CoV), and SARS‐CoV‐2.^3^ Like coronaviruses of mammalian, HCoVs are classified into alpha‐CoVs and beta‐CoVs, while the infectious viruses for birds mostly belong to the genera of gamma‐CoVs and delta‐CoVs.^4^ Among infectious strains for humans, HCoV‐229E and HCoV‐NL63 have a bat origin and are alpha‐CoVs, while HCoV‐OC43 and HCoV‐HKU1 are beta‐CoVs known to be hosted by rodents.^2^ HCoVs are important infectious agents in the etiology of both upper and lower respiratory tract infections (URTIs and LRTIs).^5^ URTIs are often caused by HCoV‐229E and HCoV‐OC43 while LRTIs are mainly linked to HCoV‐HKU1 and HCoV‐NL63 5. All these four HCoVs are low pathogenic and endemic in humans.^6^, ^7^ Besides these viruses, three CoVs, namely, SARS‐CoV, MERS‐CoV, and SARS‐CoV‐2, are highly infectious for humans, which are associated with serious health‐related problems and death.^8^

While seasonal viruses are widely distributed worldwide, their detection rate generally relies on the time and location they are active. In the current situation when SARS‐CoV‐2, the most recent and newly emerged beta‐coronavirus, is still challenging in almost all countries of the world such as Iran with millions of affected lives since early 2020, most seasonal viruses especially those targeting the human respiratory system (e.g., respiratory syncytial virus [RSV] and influenza viruses), which were active in the years just before the emergence of SARS‐CoV‐2, have mysteriously disappeared.^9^ This dramatic reduction or even delayed in typical seasons of viral circulation that is being reported by surveillance data from different countries of the world is believed to be attributable to a series of nonpharmaceutical‐mandated interventions (NPIs) (i.e., travel restrictions, borders and school closure, and hand and respiratory hygiene) used to limit the virus spread during the pandemic.^10^ In Iran, a broad range of local restrictions along with recommended measures (i.e., wearing facial masks and washing hands) for being protected against COVID‐19 have been introduced to residents immediately after the official announcement of the pandemic in early 2020.^11^

To the best of our knowledge, this study is the first national survey of HCoVs since the outbreak of COVID‐19 in Iran. Here, we collected samples and related data especially clinical information through a multicenter surveillance system of severe acute respiratory infection (SARI) during the current situation and established a quantitative RT‐PCR (RT‐qPCR) testing for seasonal HCoVs to understand the impact of the pandemic on circulation of such viruses in Iran, 2021–2022.

## 2 METHODS

### 2.1 Patients and specimen collection

To determine the impact of the pandemic on the circulation of seasonal HCoVs through 2021/2022, we designed a cross‐sectional study that was conducted during the period of October 2021 through and October 2022. About 590 throat swabs were randomly selected from respiratory samples, which were routinely screened through the national influenza surveillance by the National Influenza Center (NIC), which is located at the Virology Department, School of Public Health, Tehran University of Medical Sciences, Tehran, Iran. These samples were obtained according to the Ministry of Health (MOH) instructions from those with SARI, who visited the medical care centers for at least one of the following symptoms: High fever (>38°C) and cough with onset within the last 10 days, which requires hospitalization. All the studied samples were collected in 2–3 mL of viral transport medium, refrigerated at or below 4°C, and sent within 48 h together with a standardized test request sheet, which included patient details (e.g., age, sex, and the place of residence) and medical records (e.g., symptoms and potential risk factors such as comorbidities) from different clinics across the country (e.g., Tehran, Alborz, Arak, Ardabil, East Azerbaijan, West Azerbaijan, Bushehr, Isfahan, Fars, Golestan, Kerman, Lorestan, Razavi Khorasan, Qom, Semnan, Zanjan, and Yazd). The samples were aliquoted into two microtubes (one microtube for influenza/SARS‐CoV‐2 testing and one microtube for HCoVs) and stored at −70°C to maintain their quality intact until tested. Written informed consent was obtained from adults and the parents/guardians of included children/infants before sampling by MOH.

### 2.2 Viral genome extraction and detection

The inclusion criteria of sample selection for HCoVs testing were negative for influenza and SARS‐CoV‐2 infections. The SARS‐CoV‐2 RNA extraction kit (Viragene, Tehran, Iran) was used for ribonucleic acid isolation from the respiratory samples based on the manufacturer's protocol. After extraction, the total nucleic acids were assessed for concentration and purity by NanoDrop spectrophotometer (Thermo Fisher Scientific, USA) and a final volume of 50 μL of eluted ribonucleic acid stored in RNase free micro‐tubes at −70°C for further analysis. All samples were tested for each of the four seasonal HCoVs. SuperScript III Platinum One‐Step qRT‐PCR Kit (Invitrogen, USA) was used and PCR performed by StepOnePlus™ Real‐Time PCR Systems (Thermo Fisher Scientific, MA, USA). In brief, viral RNAs were amplified by 45 cycles of amplification in a final volume of 25 μL which was prepared as follows: 12.5 μL of 2× reaction mix, 0.5 μL of mixed primers and probe (for each virus), 0.5 units of Taq polymerase, 6.5 μL of DNase and RNase‐free water, and 5 μL of template RNA at the following thermal cycling condition: 50°C for 15 min, 95°C for 3 min followed by 95°C for 15 s, and 56°C for 30 s. For each set of reactions, negative and positive controls were included. For each of the viruses, the cycle threshold (Ct) value less than 40.0 with a typical amplification curve was considered as a detected virus. The Ct value equal to 40.0 (the suspected interval) was subjected to RT‐PCR retests.

### 2.3 Statistical analysis

Statistical analyses were performed using the R software (R version 4.2.1; 2022‐06‐23 ucrt). Pearson's chi‐squared and Fisher's exact tests were used for comparisons in terms of categorical variables. A value of *P* < 0.05 was defined as the statistical level of significance.

## 3 RESULTS

### 3.1 Demographics, prevalence, and seasonality

For over 12 months of surveillance, a total of 590 patients who complained of SARI with no confirmed influenza and SARS‐CoV‐2 infections were tested for four endemic HCoVs. As represented in Table 1, of all included patients, 341 (58%) were male and 249 (42%) were female. There were 366 (62%) and 224 (38%) with and without severe illness, respectively. Patients were categorized into five age groups, which were 0–20 (199 [33.7%]), 21–40 (130 [22%]), 41–60 (136 [23%]), 61–80 (93 [15.8%]), and >80 (32 [5.5%]). The patient's ages were between a minimum of 4 days and a maximum of 96 years with median age of 36 (IQR: 10, 57). Overall, the most common symptoms were fever and cough complained by 281/590 (48%) and 249/590 (42%), respectively.

**TABLE 1:** Characteristics of studied patients with severe acute respiratory infection.

Characteristic	*N* = 590^a^
**Inpatient and outpatient**	
Inpatient	366 (62%)
Outpatient	224 (38%)
**Age groups**	
0–20	199 (33.7%)
21–40	130 (22%)
41–60	136 (23%)
61–80	93 (15.8%)
>80	32 (5.5%)
**Sex**	
Female	249 (42%)
Male	341 (58%)
**Clinical manifestations**	
Fever	281 (48%)
Cough	249 (42%)
Sore throat	173 (29%)
Shortness of breath	163 (28%)
Muscle pain	115 (19%)
Nausea or vomiting	50 (8.5%)
Rhinorrhea	65 (11%)
Anorexia	60 (10%)
Diarrhea	35 (5.9%)
Chills	46 (7.8%)
Arthralgia	34 (5.8%)
Headache	53 (9.0%)
Chest discomfort	23 (3.9%)
Convulsion	9 (1.5%)
Malaise	8 (1.4%)
Confusion	9 (1.5%)
Restlessness	12 (2.0%)
Lethargy	7 (1.2%)
Sneezing	7 (1.2%)
HCoV‐Total	28 (4.7%)
HCoV‐229E	4 (0.7%)
HCoV‐HKU1	12 (2.0%)
HCoV‐OC43	14 (2.4%)

^a^ *n* (%); median (IQR).

From all 590 symptomatic individuals, 28 (4.7%) were positive (displaying Ct values ranging from 16 to 38) for at least one seasonal HCoVs, 16 (57%) in males, and 12 (43%) in females. However, the results showed no significant association between gender and positivity for HCoV infection (*P* > 0.05) (Table 2). In our analysis, 17 (61%) and 11 (39%) were with and without severe illness based on their requirement for hospital admission, respectively (Table 2). HCoV‐OC43 comprised half of all positive cases, detected in 14/28 (50%) (nine [64%] in males/five [36%] in females and six [43%] in inpatients/eight [57%] in outpatients) (Table 3). In addition, there were 12/28 (nearly 43%) HCoV‐HKU1 (six [50%] in males/six [50%] in females and 10 [83%] in inpatients/two [17%] in outpatients) as the second, and 4/28 (14%) HCoV‐229E (three [75%] in males/one [25%] in females and two [50%] in inpatients/two [50%] in outpatients) as the third most commonly detected virus (Tables 4 and 5). Interestingly, HCoV‐NL63 was not detected. In our survey, cases infected with HCoVs were residents throughout the country as follows: 19 Tehran, 3 Semnan, 2 Alborz, 2 Qom, 1 Ardabil, and 1 Yazd. None of the confirmed subjects suffered underlying medical conditions. The majority (>39%) of HCoV‐infected subjects were between the ages of 21 and 40 years.

**TABLE 2:** Comparison of severity of illness, sex differences, and clinical manifestations in HCoV‐infected and noninfected individuals.

Variable	Overall, *N* = 590^a^	HCoV‐Total		*P* value^b^
		No, *N* = 562^a^	Yes, *N* = 28^a^	
**Inpatient and outpatient**				0.9
Inpatient	366 (62%)	349 (62%)	17 (61%)	
Outpatient	224 (38%)	213 (38%)	11 (39%)	
**Sex**				>0.9
Female	249 (42%)	237 (42%)	12 (43%)	
Male	341 (58%)	325 (58%)	16 (57%)	
**Clinical manifestations**				
Fever	281 (48%)	263 (47%)	18 (64%)	0.071
Cough	249 (42%)	237 (42%)	12 (43%)	>0.9
Sore throat	173 (29%)	168 (30%)	5 (18%)	0.2
Shortness of breath	163 (28%)	155 (28%)	8 (29%)	>0.9
Muscle pain	115 (19%)	108 (19%)	7 (25%)	0.5
Nausea or vomiting	50 (8.5%)	49 (8.7%)	1 (3.6%)	0.5
Rhinorrhea	65 (11%)	60 (11%)	5 (18%)	0.2
Anorexia	60 (10%)	60 (11%)	0 (0%)	0.10
Diarrhea	35 (5.9%)	33 (5.9%)	2 (7.1%)	0.7
Chills	46 (7.8%)	42 (7.5%)	4 (14%)	0.3
Arthralgia	34 (5.8%)	34 (6.0%)	0 (0%)	0.4
Headache	53 (9.0%)	52 (9.3%)	1 (3.6%)	0.5
Chest discomfort	23 (3.9%)	22 (3.9%)	1 (3.6%)	>0.9
Convulsion	9 (1.5%)	8 (1.4%)	1 (3.6%)	0.4
Malaise	8 (1.4%)	8 (1.4%)	0 (0%)	>0.9
Confusion	9 (1.5%)	9 (1.6%)	0 (0%)	>0.9
Restlessness	12 (2.0%)	11 (2.0%)	1 (3.6%)	0.4
Lethargy	7 (1.2%)	7 (1.2%)	0 (0%)	>0.9
Sneezing	7 (1.2%)	6 (1.1%)	1 (3.6%)	0.3

^a^ *n* (%).

^b^ Pearson's chi‐squared test; Fisher's exact test.

**TABLE 3:** Comparison of severity of illness, sex differences, and clinical manifestations in HCoV‐OC43 infected and noninfected individuals.

Variable	Overall, *N* = 590^a^	HCoV‐OC43		*P* value^b^
		No, *N* = 576^a^	Yes, *N* = 14^a^	
**Inpatient and outpatient**				0.13
Inpatient	366 (62%)	360 (62%)	6 (43%)	
Outpatient	224 (38%)	216 (38%)	8 (57%)	
**Sex**				0.6
Female	249 (42%)	244 (42%)	5 (36%)	
Male	341 (58%)	332 (58%)	9 (64%)	
**Clinical manifestations**				
Fever	281 (48%)	273 (47%)	8 (57%)	0.5
Cough	249 (42%)	245 (43%)	4 (29%)	0.3
Sore throat	173 (29%)	170 (30%)	3 (21%)	0.8
Shortness of breath	163 (28%)	161 (28%)	2 (14%)	0.4
Muscle pain	115 (19%)	111 (19%)	4 (29%)	0.5
Nausea or vomiting	50 (8.5%)	49 (8.5%)	1 (7.1%)	>0.9
Rhinorrhea	65 (11%)	62 (11%)	3 (21%)	0.2
Anorexia	60 (10%)	60 (10%)	0 (0%)	0.4
Diarrhea	35 (5.9%)	35 (6.1%)	0 (0%)	>0.9
Chills	46 (7.8%)	44 (7.6%)	2 (14%)	0.3
Arthralgia	34 (5.8%)	34 (5.9%)	0 (0%)	>0.9
Headache	53 (9.0%)	53 (9.2%)	0 (0%)	0.6
Chest discomfort	23 (3.9%)	22 (3.8%)	1 (7.1%)	0.4
Convulsion	9 (1.5%)	8 (1.4%)	1 (7.1%)	0.2
Malaise	8 (1.4%)	8 (1.4%)	0 (0%)	>0.9
Confusion	9 (1.5%)	9 (1.6%)	0 (0%)	>0.9
Restlessness	12 (2.0%)	12 (2.1%)	0 (0%)	>0.9
Lethargy	7 (1.2%)	7 (1.2%)	0 (0%)	>0.9
Sneezing	7 (1.2%)	6 (1.0%)	1 (7.1%)	0.2

^a^ *n* (%).

^b^ Pearson's chi‐squared test; Fisher's exact test.

**TABLE 4:** Comparison of severity of illness, sex differences, and clinical manifestations in HCoV‐HKU1 infected and noninfected individuals.

Variable	Overall, *N* = 590^a^	HCoV‐HKU1		*P* value^b^
		No, *N* = 578^a^	Yes, *N* = 12^a^	
**Inpatient and outpatient**				0.15
Inpatient	366 (62%)	356 (62%)	10 (83%)	
Outpatient	224 (38%)	222 (38%)	2 (17%)	
**Sex**				0.6
Female	249 (42%)	243 (42%)	6 (50%)	
Male	341 (58%)	335 (58%)	6 (50%)	
**Clinical manifestations**				
Fever	281 (48%)	273 (47%)	8 (67%)	0.2
Cough	249 (42%)	242 (42%)	7 (58%)	0.3
Sore throat	173 (29%)	172 (30%)	1 (8.3%)	0.2
Shortness of breath	163 (28%)	157 (27%)	6 (50%)	0.10
Muscle pain	115 (19%)	113 (20%)	2 (17%)	>0.9
Nausea or vomiting	50 (8.5%)	50 (8.7%)	0 (0%)	0.6
Rhinorrhea	65 (11%)	64 (11%)	1 (8.3%)	>0.9
Anorexia	60 (10%)	60 (10%)	0 (0%)	0.6
Diarrhea	35 (5.9%)	33 (5.7%)	2 (17%)	0.2
Chills	46 (7.8%)	45 (7.8%)	1 (8.3%)	>0.9
Arthralgia	34 (5.8%)	34 (5.9%)	0 (0%)	>0.9
Headache	53 (9.0%)	52 (9.0%)	1 (8.3%)	>0.9
Chest discomfort	23 (3.9%)	23 (4.0%)	0 (0%)	>0.9
Convulsion	9 (1.5%)	9 (1.6%)	0 (0%)	>0.9
Malaise	8 (1.4%)	8 (1.4%)	0 (0%)	>0.9
Confusion	9 (1.5%)	9 (1.6%)	0 (0%)	>0.9
Restlessness	12 (2.0%)	11 (1.9%)	1 (8.3%)	0.2
Lethargy	7 (1.2%)	7 (1.2%)	0 (0%)	>0.9
Sneezing	7 (1.2%)	7 (1.2%)	0 (0%)	>0.9

^a^ *n* (%).

^b^ Fisher's exact test; Pearson's chi‐squared test.

**TABLE 5:** Comparison of severity of illness, sex differences, and clinical manifestations in HCoV‐229E infected and noninfected individuals.

Variable	Overall *N* = 590^a^	HCoV‐229E		*P* value^b^
		No, *N* = 586^a^	Yes, *N* = 4^a^	
**Inpatient and outpatient**				0.6
Inpatient	366 (62%)	364 (62%)	2 (50%)	
Outpatient	224 (38%)	222 (38%)	2 (50%)	
**Sex**				0.6
Female	249 (42%)	248 (42%)	1 (25%)	
Male	341 (58%)	338 (58%)	3 (75%)	
**Clinical manifestations**				
Fever	281 (48%)	277 (47%)	4 (100%)	0.051
Cough	249 (42%)	247 (42%)	2 (50%)	>0.9
Sore throat	173 (29%)	172 (29%)	1 (25%)	>0.9
Shortness of breath	163 (28%)	163 (28%)	0 (0%)	0.6
Muscle pain	115 (19%)	114 (19%)	1 (25%)	0.6
Nausea or vomiting	50 (8.5%)	50 (8.5%)	0 (0%)	>0.9
Rhinorrhea	65 (11%)	63 (11%)	2 (50%)	0.062
Anorexia	60 (10%)	60 (10%)	0 (0%)	>0.9
Diarrhea	35 (5.9%)	35 (6.0%)	0 (0%)	>0.9
Chills	46 (7.8%)	44 (7.5%)	2 (50%)	0.032
Arthralgia	34 (5.8%)	34 (5.8%)	0 (0%)	>0.9
Headache	53 (9.0%)	53 (9.0%)	0 (0%)	>0.9
Chest discomfort	23 (3.9%)	23 (3.9%)	0 (0%)	>0.9
Convulsion	9 (1.5%)	9 (1.5%)	0 (0%)	>0.9
Malaise	8 (1.4%)	8 (1.4%)	0 (0%)	>0.9
Confusion	9 (1.5%)	9 (1.5%)	0 (0%)	>0.9
Restlessness	12 (2.0%)	12 (2.0%)	0 (0%)	>0.9
Lethargy	7 (1.2%)	7 (1.2%)	0 (0%)	>0.9
Sneezing	7 (1.2%)	7 (1.2%)	0 (0%)	>0.9

^a^ *n* (%).

^b^ Fisher's exact test.

Notably, there was a gradual increasing trend in positivity rates of HCoVs (predominantly HCoV‐OC43 and HCoV‐229E) from fall 2021 to winter 2022 with December 2021 to February 2022 being the months with more reported cases, and a decreasing trend from spring 2022 to late summer 2022 suggesting seasonality for HCoVs. Overall, fever, cough, shortness of breath, muscle pain, rhinorrhea sore throat, and chills were the most common symptoms of 18 (64%), 12 (43%), 8 (29%), 7 (25%), 5 (18%), and 4 (14%) of all HCoVs‐infected individuals, respectively (Table 2). Among clinical manifestations, only fever was associated with HCoVs infection (*P* < 0.05), while other symptoms and gender had no statistical correlation with HCoVs infection (*P* > 0.05).

### 3.2 Co‐infections

Of the 28 HCoV‐infected individuals, mixed infections were confirmed in two patients who tested co‐positive for HCoV‐229E (Ct value: 37)/HCoV‐OC43 (Ct value: 29) and HCoV‐229E (Ct value: 35)/HCoV‐HKU1 (Ct value: 32). The former was an inpatient boy aged 12 years old who became ill on February 16, 2022, and hospitalized in Mofid children's hospital on February 19, 2022, with a high fever (>38°C) and complained of muscle pain and malaise on the first day of hospital stay. This case was previously a healthy person with no reported underlying medical condition. The latter was a 46‐year‐old male with a history of hypertension who visited a medical doctor in a local outpatient clinic in Qom, Iran on November 15, 2021, for his symptoms which were sore throat, dry cough, and chills.

## 4 DISCUSSION

As of December 27, 2022, COVID‐19 has affected 228 countries and infected more than 600 million people resulted in over 6 million deaths, worldwide (https://www.worldometers.info/coronavirus/). The disease has also been represented as a matter of concern in countries in the middle east region including Iran with 7.5 million infected and thousands of deaths since early 2020 (https://www.worldometers.info/coronavirus/country/iran/). From the very beginning of the outbreak in late 2019, mass regional and global attempts have been made to block the virus spread and to control its transmission. Like other countries, Iran has experienced a reduction in the circulation of some respiratory viruses by the introduction of strict measures and interventions (i.e., travel ban, hand hygiene, and social distances) since the first months of 2020.^12^ However, the impact of the pandemic and implemented interventions on the distribution of endemic HCoVs remained poorly determined, and data on HCoVs clinical significance, circulation, and seasonality during the pandemic are scarce. Therefore, the present study was designed to assess the detection rate of four well‐known endemic HCoVs (HCoV‐OC43, ‐NL63, ‐HKU1, and ‐229E) in a relatively large respiratory sample size obtained from patients with SARI to bring new insight into the epidemiological features of non‐SARS‐CoV‐2/noninfluenza virus infections and to determine the influence of the COVID‐19 pandemic on detection rate of endemic HCoVs in Iran.

HCoVs were found to be responsible for 4% to 5% of respiratory infections during the years after the emergence of SARS‐CoV‐2 both with respect to our and other similar studies.^13^ In accordance with recent investigations, a significant decline in HCoVs together with influenza virus infections has been observed on average during the pandemic showing that COVID‐19 strict restrictions impacted the epidemiology of non‐SARS‐CoV‐2 respiratory viruses.^14^ For instance, a letter in the Journal of Infection in 2022 reported a twofold decrease in the rate of HCoVs detection after the global emergence of SARS‐CoV‐2 compared to the prepandemic era in south of the Anatolian Peninsula in the eastern Mediterranean Sea.^14^

HCoVs in patients who have certain risk factors such as chronic cardiac or respiratory diseases and immunodeficiency are linked to hospital admission.^15^ However, this association was not observed in our investigation since no hospital‐admitted individuals with laboratory‐confirmed HCoVs had underlying medical conditions as risk factors. The results of our study showed, despite near equal detection rate, a higher infection risk for HCoVs among men compared to women with no significant differences in statistical analysis. We showed that men were more likely to be infected with HCoV‐OC43 than women, a finding that is consistent with the study by E. R. Gaunt et al.^16^ We also found that more males than females were infected with HCoV‐229E. However, in the mentioned study, equal numbers of males and females were susceptible to infection with HCoV‐229E. In a study from Iran, the majority of cases with HCoV‐229E were male, suggesting likely a sex preference for this viral infection.^17^ In the case of HCoV‐HKU1, both our patients and patients of other studies had relatively same number of men and women with no statistically sex preference.^16^

HCoVs can be detected year‐round; however, in agreement with the results of recent epidemiological studies, our analysis showed that seasonal HCoVs are more common to be detected in cold months of the winter suggesting a strong seasonal distribution for all endemic HCoVs.^18^ Levels of HCoV‐associated infections in our survey remained low throughout the summer and early fall of 2022 as similarly described by recent studies suggesting that HCoVs were not problematic during the second 6 months of 2022.^19^, ^20^, ^21^ Friedman et al. studied four endemic HCoVs in Israel between 2015 and 2016. The results of their study showed that HCoV‐OC43, HCoV‐NL63, and HCoV‐229E are at the highest level of detection in winter months; however, HCoV‐HKU1 was predominant in spring and summer seasons.^19^ Our study, however, revealed similar results for the seasonality of HCoV‐OC43 and HCoV‐229E, but did not indicate HCoV‐HKU1 a virus with common spring and/or summer circulation. In other words, HCoV‐HKU1 was most frequently detected in winter months (peaking at January). HCoV‐229E, however sporadically reported throughout the year 2021/2022, reached the highest level of detection in January with four confirmed cases. This finding was similar to the results of the study by Won Il Choi et al.^22^

HCoVs are among the most frequent respiratory viruses responsible for clinics visits for the common cold sharing a common and nonspecific set of symptoms similar to the influenza virus and also SARS‐CoV‐2 infections.^23^, ^24^ For HCoV‐OC43 and HCoV‐229E, symptoms include general malaise, headache, nasal discharge, sneezing, sore throat, fever, and cough.^3^ For HCoV‐HKU1, symptoms are fever, runny nose, cough, and shortness of breath and for HCoV‐NL63 fever, cough, rhinorrhea, tachypnea, hypoxia, and obstructive laryngitis (croup).^3^ Most of these symptoms were observed in our studied subjects regardless of the infective strain of the detected HCoVs. In our HCoVs‐infected individuals, fever, cough, and rhinorrhea were among the most common symptoms similar to the findings of other studies.^5^

As part of this surveillance, we investigated HCoV‐OC43 infection. This viral infection is generally not specified to a specific season as this virus infection was diagnosed throughout the year of 2021/2022. However, its rate of detection peaked in December and January based on our results and the results of other studies.^22^ HCoV‐OC43 was also the most predominant HCoVs in other countries like Belgium, especially in children and the elderly.^15^ A few investigations have evaluated the frequency of HCoV‐OC43 in patients clinically diagnosed with respiratory tract infections after the introduction of COVID‐19 into the nation. In recent research from Iran, of 117 respiratory samples obtained in 2020 for testing HCoV‐OC43 and SARS‐CoV‐2, 23 (20%) were confirmed for HCoV‐OC43 which is comparable to our detection rate (2.4%) in 2021/2022.^24^ This finding shows a dramatic reduction in HCoV‐OC43 abundance in 2021/2022 compared with 2020. Changes in the frequency of HCoVs in our survey might be attributed to a variation in regional multilayered health‐related measures and differences in geographical locations with different weather conditions and socioeconomic status. In mentioned research similar to ours, most of the HCoV‐OC43 patients had fever and cough as two chief clinical manifestations.^24^

HCoV‐HKU1 was reported to be the cause of 0.04% to 2.1% of adult respiratory diseases around the world,^25^ and also in Iran based on our analysis, which was detected in 2% of studied subjects with a respiratory infection. HCoV‐HKU1 was found as both a single and mixed with other respiratory viruses in studies from Iran.^26^ In our investigation, similar to other studies, respiratory symptoms were the most common clinical manifestations of HCoV‐HKU1; however, nonrespiratory involvements are also described such as gastrointestinal symptoms (e.g., diarrhea).^25^ Most of the HCoV‐HKU1‐infected cases in our study (10 out of 12 virus‐infected cases) were hospitalized for the severity of their illness. In agreement with this finding, there is some evidence regarding the requirement of oxygen and intensive care beds for critical care of patients with HCoV‐HKU1 infection showing that a general belief that HKU1 causes only mild respiratory infections are not true all the time.^25^ HCoV‐HKU1‐caused severe respiratory diseases, however, might be rare, and it can occur in immunocompromised and/or even sometimes in immunocompetent individuals.^27^, ^28^ Our virus‐infected cases had no history of underlying diseases to discuss.

As another endemic HCoV, we investigated HCoV‐NL63. HCoV‐NL63 was first detected in Iran in 2012 in a newborn girl who had suffered from respiratory distress.^29^ Since the first detection, the virus infection has been diagnosed in a few studies across the nation.^23^, ^26^ An investigation from Iran (before the pandemic) in a similar duration time as our study found HCoV‐NL63 by using the same method as our method in nasal and throat swabs of 23.9% of cases (specified children aged <5 years) who had acute respiratory infections.^23^ Given the differences between the inclusion criteria of this study compared with ours, different results could be explained. In addition to this study, there were some pieces of evidence from different countries with a lower detection rate (for instance, 1.2% in Japan,^30^ 2.1% in Australia,^31^ and 2.5% in Canada^32^), regarding the virus infection. Here, we found no HCoV‐NL63 in any of the samples during the study period (even in winter season when the virus is in highest detection rate in many countries^33^) contrary to what has been detected in the years just before the beginning of the pandemic in Iran^23^ and other parts of the world.^5^ Of note, this finding is not surprising because other national surveillance before the pandemic with smaller sample sizes than ours^17^ or by different methods of detection as our used method^34^ found no evidence of HCoV‐NL63 in studied cases with respiratory infections. HCoV‐NL63 is more common in children; however, in our study, none of the children was diagnosed with this virus infection, which is consistent with reports outside Iran.^33^ Given the fact that changes in the epidemiological pattern of common respiratory viruses had been observed and have already been described even in recent months in the course of the COVID‐19 pandemic,^12^ we presumed that COVID‐19 could have impacted the transmission dynamics of HCoV‐NL63 during 2021/2022, and the implementation of NPIs used against viral surge has successfully controlled the activity of HCoV‐NL63 throughout the 2021–2022 seasons; however, it needs to be well established. While we are not sure about the NPIs responsible for this observation, a phenomenon called “viral interference” might also be a reason of why HCoV‐NL63 was not detected in this research; however, further studies are required for this to be well determined. In addition, HCoV‐NL63 and SARS‐CoV‐2 were found to share recognized epitopes by the B cell‐mediated immune response; thus, it is not clear if previous exposure to SARS‐CoV‐2 conferred a protective immunity against HCoV‐NL63; a hypothesis has been supported in recent research.^35^

Coronaviruses can be found in simultaneous viral infections whether with other viruses or especially viruses in the same viral classification.^36^ Lu R et al. screened a total of 981 nasopharyngeal swabs taken from adults in Beijing, China, from 2009 to 2011 for respiratory viral infections. All included subjects had acute URTIs. Real‐time RT‐PCR was used for viral detection in these patients. The detection rate for HCoVs was estimated 16.0% (9.8% for HCoV‐229E, 4.3% for HCoV‐OC43, 1.6% for HCoV‐HKU1, and 1.1% for HCoV‐NL63). There were seven HCoVs‐positive samples co‐positive for at least one additional HCoV, which were three samples for HCoV‐OC43/HCoV‐229E, one sample for HCoV‐OC43/HCoV‐229E/HCoV‐NL63, one sample for HCoV‐OC43/HCoV‐HKU1/RSV, one sample for HCoV‐NL63/HCoV‐HKU1/rhinoviruses, and one sample for HCoV‐OC43/HCoV‐NL63/rhinoviruses.^37^ In a recent survey from Iran, the authors co‐detected (HCoV‐229E and HCoV‐HKU1) and (HCoV‐NL63 and HCoV‐HKU1) in their included subjects.^26^ A report from Thailand showed young children with acute LRTI whose nasopharyngeal secretions were tested for HCoVs which infected with HCoV‐229E (3.54%) and HCoV‐OC43 (0.88%) and one sample was tested co‐positive for these viruses.^38^ In line with the above findings, analysis of HCoV strains co‐infectivity revealed that two (7.14%) out of the total number of our studied cases were co‐infected with viral strains, one patient had HCoV‐OC43 and HCoV‐229E mixed infection, and the other one had dual infection with HCoV‐HKU1 and HCoV‐229E. As represented here and similar to the results of other studies,^15^ HCoV‐229E is common in our patients who were diagnosed with two types of HCoVs.

This study has some limitations. First, we worked only on influenza and SARS‐CoV‐2 tested negative specimens. This may increase the likelihood of missing detection of HCoV co‐infections with such co‐circulating respiratory viruses. Second, we performed our analysis on only throat swab samples. Considering the potential of HCoVs for infection of lower respiratory tracts,^16^ if samples such as sputum or bronchoalveolar lavage fluid were used, the positivity rate of HCoVs could be changed. Third, given the fact that NIC receives and tests clinical samples from different parts of the nation, if proper sampling procedure and transportation were underestimated, viruses may be lost resulting in no virus detection. Thus, findings especially regarding HCoV‐NL63 should be interpreted with caution. Finally, infection with HCoV‐OC43, HCoV‐NL63, HCoV‐HKU1, and HCoV‐229E are sometimes symptoms free^39^; hence, by analyzing only symptomatic patients, we missed data regarding the disease burden due to HCoVs infection.

Here, we highlighted the importance of seasonal HCoVs (especially HCoV‐OC43) in the etiology of non‐SARS‐CoV‐2/noninfluenza viruses and brought attention to consider such viruses as circulating pathogens among patients with respiratory infections in Iran in 2021/2022. More investigations are needed to find more about the circulation pattern of endemic HCoVs, the impact of COVID‐19, and its burden on the activity and seasonality of non‐SARS‐CoV‐2 coronaviruses and to detect additional pathogens in CoV‐infected individuals during the COVID‐19 pandemic.

## AUTHOR CONTRIBUTIONS

**Hassan Karami**: Writing the manuscript. **Kaveh Sadeghi**: Data curation. **Sevrin Zadheidar**: Investigation. **Fatemeh Saadatmand**: Data collection. **Negar Mirsalehi**: Methodology. **Nima Hoveidi Ardestani**: Data collection. **Shirin Kalantari**: Methodology. **Mohammad Farahmand**: Data analysis. **Jila Yavarian**: Editing. **Talat Mokhtari‐Azad**: Supervision.

## CONFLICT OF INTEREST STATEMENT

The authors declare no conflicts of interest.

## ETHICS STATEMENT

Written informed consent was obtained from all patients by MOH.

### PEER REVIEW

The peer review history for this article is available at https://publons.com/publon/10.1111/irv.13128.

## PATIENT CONSENT STATEMENT

Written informed consent was obtained from all patients as the samples were taken for influenza surveillance via MOH.

## Data Availability

The data that support the findings of this study are available from the corresponding author upon reasonable request.
